# A CNN-Transformer Fusion Model for Proactive Detection of Schizophrenia Relapse from EEG Signals

**DOI:** 10.3390/bioengineering12060641

**Published:** 2025-06-12

**Authors:** Sana Yasin, Muhammad Adeel, Umar Draz, Tariq Ali, Mohammad Hijji, Muhammad Ayaz, Ashraf M. Marei

**Affiliations:** 1 Department of Computer Science, University of Okara, Okara 56300, Pakistan; sana.yasin@uo.edu.pk (S.Y.); adeelrasheed02@gmail.com (M.A.); 2 Department of Computer Science, University of Sahiwal, Sahiwal 57000, Pakistan; 3 Artificial Intelligence and Sensing Technologies (AIST) Research Center, University of Tabuk, Tabuk 71491, Saudi Arabia; 4 Faculty of Computers and Information Technology, University of Tabuk, Tabuk 71491, Saudi Arabia; m.hijji@ut.edu.sa

**Keywords:** unimodal data, robustness, transformer model, schizophrenia disorder, personalized relapse prevention

## Abstract

Proactively detecting schizophrenia relapse remains a critical challenge in psychiatric care, where traditional predictive models often fail to capture the complex neurophysiological and behavioral dynamics preceding recurrence. Existing methods typically rely on shallow architectures or unimodal data sources, resulting in limited sensitivity—particularly in the early stages of relapse. In this study, we propose a CNN-Transformer fusion model that leverages the complementary strengths of Convolutional Neural Networks (CNNs) and Transformer-based architectures to process electroencephalogram (EEG) signals enriched with clinical and sentiment-derived features. This hybrid framework enables joint spatial-temporal modeling of relapse indicators, allowing for a more nuanced and patient-specific analysis. Unlike previous approaches, our model incorporates a multi-resource data fusion pipeline, improving robustness, interpretability, and clinical relevance. Experimental evaluations demonstrate a superior prediction accuracy of 97%, with notable improvements in recall and F1-score compared to leading baselines. Moreover, the model significantly reduces false negatives, a crucial factor for timely therapeutic intervention. By addressing the limitations of unimodal and superficial prediction strategies, this framework lays the groundwork for scalable, real-world applications in continuous mental health monitoring and personalized relapse prevention.

## 1. Introduction

Schizophrenia is a complex mental illness that continues to pose a major challenge to modern psychiatry. Despite decades of research, long-term effective management remains elusive due to recurrent psychotic episodes and complex symptom dynamics [1]. Originally defined by Emil Kraepelin and later recharacterized by Eugen Bleuler, the disorder involves intermittent breaks with reality, altered cognition, and impaired social functioning. It affects more than 24 million people globally—approximately 1 in 300—while adult prevalence reaches 0.45%. Alarmingly, more than half of diagnosed patients relapse within one year of discontinuing medication, exposing critical gaps in predictive treatment strategies [2]. Recent advances in deep learning provide promising tools for modeling the detailed neurophysiological and behavioral patterns associated with recurrence [3]. In addition, online platforms and patient communities provide valuable information on real-world factors, including substance use, emotional triggers, and side effects of medications [4]. However, these unstructured data sources require careful handling to ensure reliability and patient privacy. By integrating clinical metrics with digital behavioral information, researchers can develop more accurate and personalized recurrence prediction systems, paving the way for timely interventions and improved psychiatric care [5].

**Figure 1:**
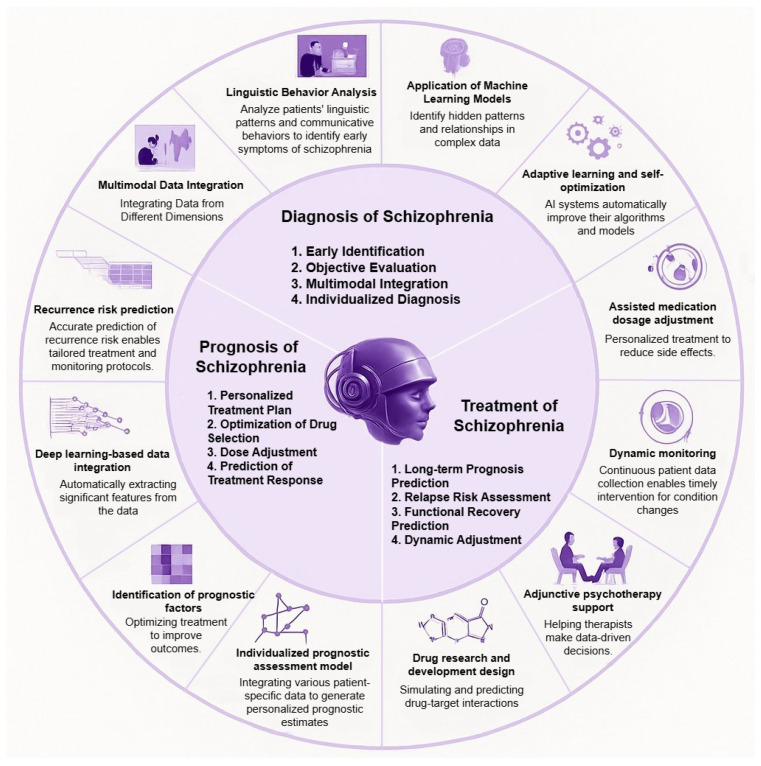
Conceptual framework that features essential AI-driven elements for schizophrenia diagnosis and treatment and prognosis. The diagram combines multimodal data analysis with deep learning and clinical support features to support early detection and individualized treatment alongside proactive relapse prevention.

Figure 1 demonstrates the application of AI tools like deep learning and multimodal integration to support schizophrenia care throughout diagnosis, prognosis, and treatment. The methodology focuses on linguistic behavior analysis together with data fusion techniques and adaptive decision support systems to enhance predictive modeling and thereby increase accuracy and personalization in clinical applications. Precision psychiatry benefits from these elements because they facilitate early prediction of relapses while optimizing medication and supporting real-time monitoring. The primary objective of this study is to establish an accurate prediction model for relapse in schizophrenia through using electroencephalogram (EEG) recordings and innovative deep learning (DL) techniques. The framework consists of important components including the data preprocessing, the feature extraction, and a hybrid Transformer-based deep learning network, which enables it to efficiently learn the complex neuronal patterns. Moreover, the latter study highlights the need to establish preventive measures, including early intervention practices—i.e., psychiatric screenings on a regular basis and predictive monitoring—and personalized adjustments of treatment options: medications (dose optimization) and personalized therapeutic strategies [6].

Additionally, inclusion of social media trend analysis combined with real-time information from wearables provides a comprehensive view of how the mental health of patients is affected in a dynamic, real-world scenario. This synergism not only provides better predictive accuracy of relapse, but also indirectly enhances a proactive and continuous care model for care in schizophrenia [7]. Together, the presented framework aims to improve patient outcomes, reduce relapse rates, and contribute to the growing shift toward personalized data-driven mental health solutions.

To achieve the above-stated objectives, we developed a hybrid deep learning model that combines Convolutional Neural Networks (CNN), Transformer architectures, flattened layers, and fully connected layers to identify potential markers for predicting risk of schizophrenia relapse [8]. In the first modelling paradigm, EEG is combined with sentiment analysis and with clinical assessment tools including Positive and Negative Syndrome Scale (PANSS), Brief Psychiatric Rating Scale (BPRS), Global Assessment of Functioning (GAF), and Montgomery–Åsberg Depression Rating Scale (MADRS), all of which are important for the clinical progress of the disorder [9]. The data acquisition was carried out from electronic health records and structured clinical interviews, leading to a high-quality and very comprehensive dataset [10].

One important advantage of this approach is that it enables personalized treatment strategies addressing individual risk factors with specific interventions to enhance treatment outcome [11]. However, challenges remain in terms of data quality, ethics of data use, and limited coherent research frameworks [12]. Although social and behavioral data are often referenced, the use of these data to inform predictive modeling is still relatively low as studies have often relied heavily on clinical indicators. This study aims to fill that gap by integrating social media-derived data and clinical parameters to generate an improved relapse prediction model [13]. By leveraging such an interdisciplinary approach, we hope to advance a data-driven framework for the provision of mental health care. Our Transformer-based model—which aims for a predictive accuracy of more than 90%—offers a high reliability for forecasting schizophrenia relapse.

Clinical and neural data analysis with Transformer-based deep learning models demonstrates potential for schizophrenia relapse prediction through long-range temporal dependency detection [14]. These models excel at anomaly detection compared to traditional methods but face challenges because they need extensive labeled data and struggle with small noisy EEG signals [15]. Their application to MRI data analysis has shown essential brain region interactions, yet the spatial resolution alone does not capture relapse dynamics [16,17]. The blending of imaging data with clinical and demographic information leads to better predictive abilities [18] but current models fail to account for essential sentiment cues and behavioral patterns necessary for relapse identification. Transformer models with multi-head attention effectively process various input types yet they do not present understandable ways to connect different modalities [19]. Graph-based frameworks alongside attention-driven systems achieve enhanced robustness [20] yet encounter challenges with personalized and generalized patient treatment approaches.

The limitations of schizophrenia-specific applicability persist despite transfer learning and data augmentation addressing training scarcity [21] and large-scale datasets like HCP pretraining neuro models [22] because of domain shifts and demographic mismatches. Federated learning supports privacy-conscious model sharing between institutions [23] yet faces technical challenges when incorporating EEG and temporal data sequences in this framework. The clinical applicability of these models suffers due to their high complexity and substantial computational requirements [24]. Rule-based hybrid models provide interpretability [25] while struggling to adjust to patient condition changes. Model output associations with clinical risk factors improve interpretability [26], though these rule-driven mappings frequently reduce complex neural phenomena to simple models. Federated learning provides potential for fairness and inclusiveness [27,28,29], yet we have yet to determine its usefulness for real-time EEG analysis involving multiple modalities. While attention visualization and counterfactual reasoning have enhanced trust in deep learning predictions [30], few models apply these XAI methods to temporal frameworks specific to relapse prediction. VAEs and GANs address data distribution imbalances yet risk producing unrealistic artifacts and altering neurophysiological distributions [31,32]. Self-supervised learning enables the extraction of useful information from unlabeled data [33,34] but current methods do not adequately address the specific needs of dynamic psychiatric conditions.

The latest hybrid architectures that combine CNNs with Transformers provide improved spatial-temporal feature extraction [35,36] yet fail to incorporate contextual behavioral data. The current frameworks fail to adequately address ethical issues related to fairness and transparency. The healthcare field urgently requires a model that integrates multiple modalities while ensuring clinical relevance and interpretability. We present a CNN-Transformer fusion framework to learn spatial-temporal EEG patterns and combine them with sentiment analysis and clinical scores while improving minority-class learning through SMOTE. Our model architecture uniquely integrates high performance with interpretability to deliver a scalable solution for proactive schizophrenia relapse prediction [37,38,39,40,41]. Recent research confirms that deep learning systems such as neural networks and hybrid approaches are effective for diagnosing psychiatric disorders through EEG analysis for conditions including major depressive disorder and bipolar disorder as well as neurological symptoms associated with COVID-19 [42,43,44]. The research demonstrates EEG’s expanding application as a non-invasive scalable biomarker for mental health through active and passive EEG data integration. Our study advances this research area by utilizing a multimodal CNN-Transformer model to predict schizophrenia relapse through EEG signals and clinical plus sentiment-derived features.

**Figure 2:**
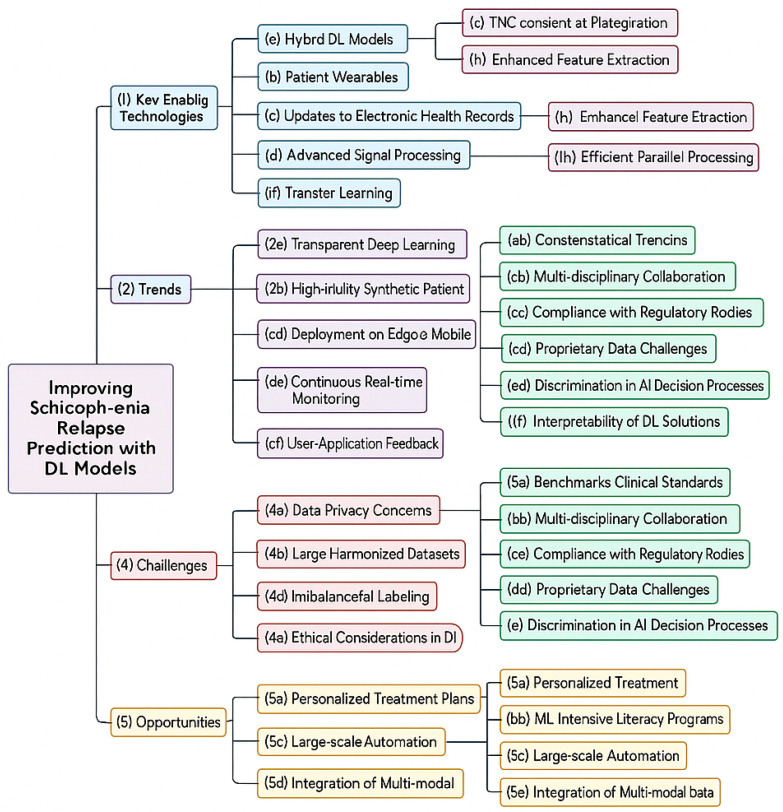
Conceptual mind map outlining the enabling technologies, emerging trends, challenges, and opportunities in optimizing schizophrenia relapse prediction through deep-learning (DL) models. The diagram organizes key factors into five distinct categories: (I) Key Enabling Technologies, (II) Trends, (III) Challenges, (IV) Opportunities, and (V) Ethical and Infrastructure Considerations. Each category further highlights specific subdomains relevant to clinical integration, data management, and intelligent health care delivery.

Figure 2 presents a comprehensive flowchart of the technologies, trends, challenges, and opportunities related to improving schizophrenia relapse prediction using DL models. It outlines key enabling technologies like hybrid DL models, data preprocessing, and sentiment analysis integration. The trends section focuses on advancements such as real-time monitoring, data fusion, and federated learning for global collaboration. Challenges discussed include data privacy concerns, class imbalance, and high computational costs. Lastly, the opportunities section emphasizes personalized treatment plans, early detection, and the integration of multimodal data to enhance prediction accuracy and clinical outcomes.

This research tackles existing challenges by deploying a fusion model combining CNN and Transformer architectures to analyze both spatial and temporal EEG signal dependencies. Our design includes sentiment analysis and clinical scores to establish a strong multimodal prediction framework. The research study focused on resolving class imbalance by employing SMOTE alongside augmentation strategies which resulted in enhanced sensitivity metrics and reduced false negatives.

The study presents a deep learning framework which provides strong and clinically meaningful solutions to predict early schizophrenia relapses. This research presents a fundamental methodological advancement which uses a hybrid deep learning model that merges Convolutional Neural Networks (CNNs) for extracting spatial information with Transformer models to detect long-term temporal patterns in EEG signals. The combined architecture allows the model to perform neural dynamics analysis more effectively compared to single architecture systems. The integration of various data sources such as EEG signals and clinical scales PANSS and BPRS with sentiment-derived features into one prediction pipeline represents another essential contribution. A multidimensional perspective strengthens both the contextual understanding and diagnostic accuracy of the model. The study addresses class imbalance by applying Synthetic Minority Over-sampling Technique (SMOTE) and EEG-specific augmentation methods to enhance sensitivity while decreasing majority class bias. The empirical evaluations produced prediction accuracy results of 97% along with notable improvements in recall and F1-score metrics. The model’s clinical usefulness in planning proactive treatment becomes evident through its decreased number of false negatives. These elements together create an interpretable monitoring system that supports personalized treatment while providing scalable schizophrenia surveillance. Our study combines behavioral and medication-related features with clinical AI principles to enhance relapse prediction accuracy in real-world applications. We incorporate findings from Besana et al. to reinforce the clinical framework of our research. It completed a multicenter retrospective analysis to discover primary readmission predictors in young adults experiencing their initial episode of psychosis. According to their research findings, long-acting injectable antipsychotics (LAIs) decreased relapse risk whereas substance use raised the chances of hospital readmission. Our model’s emphasis on adherence and behavioral and affective indicators is reinforced by these findings and our approach gains validation through a clinical framework. Our predictive system gains enhanced translational value through this connection which aligns our feature space with known psychiatric risk markers [41].

Table 1 presents a comparison of existing schizophrenia prediction methods while detailing their objectives, models, accuracy levels and the types of data used. The listed studies identify the proposed hybrid model (CNN + Transformer + Sentiment Analysis) as the most effective because it achieves 97% accuracy while enabling early detection through EEG data.

## 2. Material and Methods

The primary objective of this study is to develop a reliably predictive model for the early detection of schizophrenia relapses by leveraging a combination of advanced DL techniques. The proposed approach integrates CNN and Transformer models to enhance predictive accuracy and improve early diagnosis. Early detection of schizophrenia relapses is crucial in clinical settings as it enables timely intervention, reduces the severity of symptoms, and improves patient outcomes. Combining different DL models, the study aims to exploit their strengths to achieve superior predictive performance.

**Figure 3:**
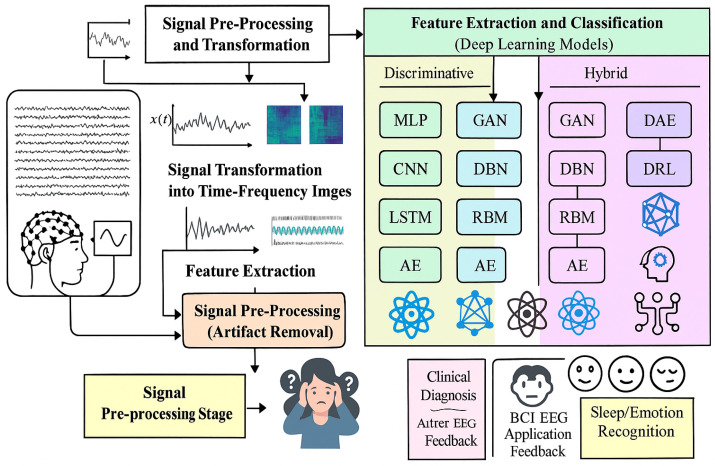
EEG signal processing pipeline in relation to mental health prediction. It shows the workflow from EEG raw acquisition and pre-processing to time-frequency and feature extraction. The listed features are then used by discriminative models (SVM, LDA, RF, GRU) and deep learning models (GAN, DBN, CNN, LSTM, GRU) for classification to assist in clinical diagnosis, BCI applications, and neuroergonomics.

Figure 3 shows a complete pipeline of EEG signal processing, starting from collection of raw brain signal, which is further pre-processed and transformed in order to improve the quality of signal. These are the steps after we convert signals to time-frequency representations and extract meaningful features. The features are then fed either into the classical discriminative models such as SVM, LDA, RF, and GRU, or the deep learning models including GAN, DBN, CNN, LSTM, and GRU, for classification. These ultimate results can be beneficial for multiple applications ranging from clinical diagnosis to BCI systems, neuroergonomics and other EEG-based assessments facilitating advanced mental health monitoring and cognitive assessment.

### 2.1. Data Collection and Understanding

The dataset used in this study includes patients diagnosed with schizophrenia and healthy control’s reliability in addition to EEG recordings. As time-series data, EEG data brings various challenges, such as noise artifacts, missing values, and variability in signal quality across different subjects. Since EEG signals are crucial in studying the brain, they must have high integrity and reliability. This track will highlight the latest innovations in EEG data integrity and reliability. To overcome these issues, a complete data pre-processing pipeline was developed to clean up, normalize, and improve the quality of the recorded signals. This pipeline was adapted for noise reduction, treating missing values, and scaling signal amplitudes to keep a reliable and increased measurement process dataset. This was followed by an EDA phase to better understand the dataset and its intrinsic characteristics. In this phase, the statistical distribution of the data was used to explore the dataset for anomalies and to visualize EEG waveforms across multiple channels to identify inconsistencies or abnormalities. Combining descriptive statistics and graphical techniques (histograms, box plots, line plots), we assessed key features of the dataset, including signal amplitude mean, signal variance across electrode sites, and the presence of artifacts (which could bias the results). These initial findings enabled a holistic understanding of the data, forming the subsequent features of the engineering and pre-processing steps. Additionally, signal quality was assessed, removing recordings that did not align with the requirements for reliable analysis. Several filtering techniques were performed to remove powerline interference, muscle artifacts, and baseline drifts to preserve the reliability of the signals. Common missing points (due to transient signal loss or electrode detachment) from EEG recordings were handled by imputation methods (interpolation or model-based estimations). This provided a continuous, coherent dataset and reduced the likelihood of artifacts associated with the incomplete recordings. Cross-validation methods were used to evaluate the reproducibility of EEG signatures across subjects. Using spectral and temporal dynamics, differences between schizophrenia patients and healthy controls were systematically investigated. This enabled better target feature engineering in prediction and classification tasks. The results of the EDA segment provided a basis for customizing the preprocessing approaches, enhancing the effectiveness and dependability of the downstream DL models utilized in this research. This systematic data collection and understanding process helped us prepare the dataset for the following analytical methods. The steps taken in this phase to preprocess and analyze the data were integral in increasing the validity of the findings, ensuring that the study results were based on a strong data foundation.

We collected EEG data through a BioSemi ActiveTwo system equipped with 64 channels and a sampling rate of 256 Hz. The study received ethical approval from the institutional review board after obtaining informed consent from all participants. The collection contains EEG recordings of 85 individuals including 42 schizophrenia patients and 43 healthy controls and features gender parity with 46% female participants and 54% male participants aged between 19 and 55. The EEG acquisition protocol combined measurements of the resting state with task-based stimuli. The raw data were processed in the 0.5 to 50 Hz range followed by Independent Component Analysis (ICA) processing to eliminate eye and muscle artifacts. We cannot share raw data due to privacy protection protocols, but we will release anonymized features and model code upon acceptable request to support reproducibility.

### 2.2. Handling Missing and Duplicate Values

Missing data is one of the major obstacles in the processing of EEG data, where sensor malfunctioning, movement artifacts, data collection, etc, cause the issue. Appropriate handling of missing values is crucial since random missing values can lead to considerable biases, skew statistical analyses, and compromise the precision and reliability of DL models [39]. To resolve this, we adopted a systematic and organized methodology to preserve the data’s integrity and prevent any inaccurate analysis thereafter. Dealing with missing values in the dataset required finding out what percentage of the data was missing. Records with a high presence of NaN values were detected and dropped. This step aimed to smooth out the influence of these records on the model training, avoiding noise, bias, or misleading patterns. Through the removal of overly incomplete records, the dataset was retained in such a way as to preserve robustness and representativeness for modeling and analysis. In cases where data was missing only in individual channels or at selected time points, suitable imputation methods were utilized to ensure data continuity. We used common imputation techniques, namely mean and median imputation, where the mean/median of the corresponding channel would replace missing data. Introducing synthetic data through imputation techniques, such as mean or median imputation, allowed the retention of information while reducing variance between the two datasets. Beyond simple imputation methods, interpolation techniques were used to reconstruct missing time series data using underlying temporal trends. Missing values were addressed using linear interpolation, which assumes a straight-line relationship between known data points. This considers data up to 20 elements, making approximations whereas cubic interpolation provides more advanced and smoother estimates by considering how curves are between things. The interpolation approaches enabled a more accurate recovery of missing information while preserving the natural variation in EEG signals. In addition to dealing with missing values, the dataset was checked for duplicate records that could add redundancy and distort analysis outputs. We then detected and eliminated duplicates, which helped minimize distortions in our dataset by preventing multiple entries with the same EEG signal. It was an essential step to maintain the data. Was not everyone made to be overrepresented in any particular distribution from such model training? After preprocessing with compression (removing duplicates) and changing text formats, the data was used to retain only complete observations that were accurate and reliable. This dataset-cleaning method resulted in a cleaner dataset and ensured the validity of subsequent analyses and model-building processes on the EEG data.

### 2.3. EEG Preprocessing: Noise Removal and Signal Enhancement

We applied preprocessing to our EEG data using EEGLAB toolbox version 2023.0 within MATLAB R2022b to maintain consistent and reproducible results for all recordings [44]. To minimize low-frequency drift and high-frequency muscle artifacts, the raw EEG data underwent initial band-pass filtering through a 4th-order Butterworth filter which set cutoff frequencies between 0.5 Hz and 50 Hz. The recordings underwent additional processing with a 50 Hz notch filter to remove power line noise. EEG channels with abnormal amplitudes or poor signal quality automatically received flags when a z-score threshold was exceeded and then underwent interpolation with spherical spline techniques. The FastICA algorithm executed Independent Component Analysis (ICA) to eliminate artifacts and set the number of independent components to match the 64 recorded EEG channels. Researchers identified artifact-related components such as eye blink signals, ocular movement patterns, muscle tension traces, and cardiac rhythm traces using spatial topography analysis in combination with temporal waveform observation and auxiliary EOG channel correlations followed by manual expert verification. We excluded these identified components prior to rebuilding the cleaned EEG signals for further analysis. The preprocessing strategy established a balance between robust artifact removal and neural signal preservation to create a clean dataset appropriate for deep learning-based relapse prediction.

We confirmed that all preprocessing steps maintained the full rank of the EEG data matrix before performing ICA decomposition to ensure its validity. Our preprocessing avoided any dimensionality reduction and rank-deficient interpolation along with aggressive referencing which would otherwise damage the data structure. This validation shows that the ICA algorithm processed a full-rank matrix which enables accurate source separation along with dependable artifact removal. The discussed practice matches the best-practice guidelines in [41] which highlight the critical role of preserving data rank for optimal ICA performance and preventing feature distortion.

### 2.4. Feature Extraction and Normalization

Once the EEG signals were cleaned and refined, meaningful representations were extracted from the raw data. This latter procedure was key for using less complicated signals without losing important content for detecting schizophrenia. The statistical characteristics were calculated for all channels in the EEG data, allowing the model to learn the basic distributional aspects of the EEG signals. Representations: Descriptive features included mean, standard deviation, skewness, and kurtosis, which provided insight into the central tendency, dispersion, and shape of the EEG signal distribution. Such features facilitated the quantification of differences in cortical activity and identifying possible anomalies related to schizophrenia. In addition to statistical features derived from APR, techniques from the frequency domain, such as the Fourier Transform, were applied to derive relevant features. Inspired by the Fourier Transform, we implemented this decomposition to isolate the various frequency components of EEG signals and analyze spectral patterns likely associated with neurological disorders. Power spectral density (PSD) was calculated to evaluate signal energy distribution in different frequency bands. Because different frequency bands (e.g., delta, theta, alpha, beta, gamma) are related to other cognitive and mental states, examining their spectral properties yielded additional insights into the neural properties that underlie schizophrenia. The combination of features gave the model a more complete description of the EEG signals, improving the model’s performance in classifying healthy and affected individuals. Due to the high variability of the EEG signal amplitudes between individuals and recording conditions, normalization was applied to make the extracted features consistent and comparable. Normalization is a crucial preprocessing step because EEG signals can vary significantly across individuals due to differences in electrode placement, scalp conductivity, and other physiological factors. These include two popular normalization methods: min-max normalization and Z-score normalization. The normalization technique used was a min-max normalization, which scales the features up to a fixed range (always between 0 and 1), allowing differences to remain while ensuring numerical stability. Conversely, Z-score normalization standardized the characteristics to conform to a standard normal distribution, with a mean of 0 and standard deviation of 1, reducing the effect of outliers and signal amplitude variation. Such normalization approaches made the dataset homogeneous, minimizing biases introduced due to amplitude variation and enabling DL to learn from a more consistent feature space. All of this enhanced the generalizability and robustness of the classification models, which helped detect schizophrenia using EEG signals, which were much more accurate and reliable.

### 2.5. Encoding Categorical Labels and Data Splitting

Label encoding was used to convert categorical class labels (schizophrenia and healthy control) into numerical representations in this study. As most algorithms require numerical input instead of categorical labels, this transformation was necessary to train DL models effectively. This process helped ensure that the respective classification algorithms could interpret the data accordingly by converting categorical labels to numerical values. In addition, label encoding allowed for the maintenance of the interpretability of the data and ensured compatibility with many classification methods for a smooth and effective modeling process. Subsequently, after the dataset is encoded, the dataset is divided into training and testing sets in an 80-20 ratio. This separation was performed in such a way as to ensure that the proportions of schizophrenia and healthy controls stayed the same in each part. This stratification was aimed primarily at avoiding a potentially damaging data imbalance that could offset the model’s performance. This increases the likelihood that the model will generalize well to unseen data because it adequately represents both categories in training and testing sets. By implementing this method, the model was less likely to develop a bias towards the majority class, improving its accuracy and trustworthiness when making predictions. Since the dataset contained an imbalance in class distribution, the Synthetic Minority Over-sampling Technique (SMOTE) was used to treat class imbalance further. One more advanced resampling technique is named SMOTE, which generates synthetic samples for the minority class, which means that, in our case, schizophrenia relapse cases, SMOTE performed an over-sampling algorithm to increase the representation of the minority class by adding artificial data points to reduce bias caused by class imbalance. Overall, this helped ensure that the model did not overly prefer the classes that dominated the dataset, improving overall classification accuracy and robustness. In this regard, the application of SMOTE was especially helpful, as the number of schizophrenia relapses in the dataset was inherently limited. Without proper weighting, the model could have poorly learned the patterns corresponding to these cases, resulting in undesirable performance. Synthetic samples improved the training process by allowing the model to understand schizophrenia relapse patterns better. In this way, we improved the classification accuracy, and, at the same time, we ensured that the model was fair and effective even during real-time prediction.

**Figure 4:**
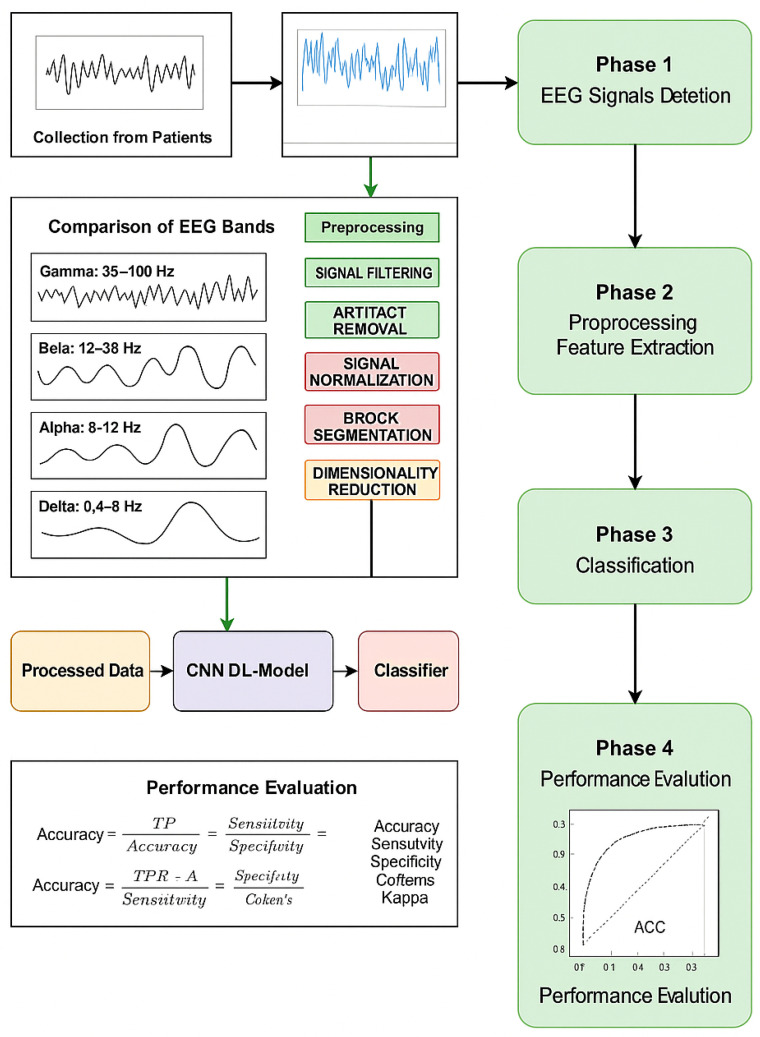
Workflow of EEG-based schizophrenia relapse prediction using a CNN-Transformer hybrid model.

Figure 4 presents the four-phase deep learning pipeline to predict schizophrenia relapse from EEG signals, including EEG signal acquisition from subjects (Phase 1, the first step). Phase 2 includes preprocessing stages (e.g., signal filtering, artifact removal, normalization, epoch segmentation, feature extraction, and dimensionality reduction). In phase 3, a CNN-based deep learning model is used to classify the processed data. The fourth phase conducts a performance evaluation using the main metrics such as precision, sensitivity, specificity, F1 score, and Cohen’s Kappa coefficient.

### 2.6. Addressing Class Imbalance and Data Augmentation

Class imbalance is a common challenge in medical datasets, especially in the case of neurological and psychiatric disorders. When one category (e.g., healthy controls) is over-represented in the dataset compared to another category (e.g., subjects diagnosed with schizophrenia), such an imbalance may result in biased model performance, where the model favors the majority class but has lower prediction rates for minority class instances. Such bias can be detrimental in clinical applications, where it is important to classify affected individuals accurately for diagnosis and treatment planning. SMOTE was used to become aware of this problem and for more balanced data distribution regarding classes. The SMOTE technique is a sophisticated resampling algorithm that fights class imbalance by generating synthetic samples for the underrepresented class (rather than replicating the labels of existing instances). This is done using interpolation of new samples based on feature-space similarities between the existing examples of the minority class. As our synthetic data points reflect the original training samples, the SMOTE helps prevent overfitting and ensures that our model is trained on a balanced dataset. As a result, classification performance is enhanced, and the model can better predict, especially for minority cases. Including SMOTE in the preprocessing pipeline helped ensure that the dataset was more representative of real-world conditions and improved the model’s ability to distinguish between different classes more effectively. Moreover, data augmentation techniques were utilized to enhance this model’s robustness and generalizability, in parallel with addressing the class imbalance. As EEG signals are highly variable and susceptible to noise, special augmentation strategies are needed to enhance the classier models’ performance. Gaussian noise was added to EEG signals to add natural variability to the dataset. This mimicked real-world distractions, like electrical interference or physiological noise, exposing the model to more varied training instances. Introducing Gaussian noise, the model gained tolerance to small perturbations, resulting in better generalization across varying EEG recordings. Additionally, temporal-shift approaches were applied to change the temporal characteristics of the EEG signal. New variants can be introduced by making a time-wise shift of the signal sequences without modifying the major aspects of the data. This approach expanded the training set, so the model saw mutants with signal patterns that it did not see during training. Moreover, long EEG series were divided into overlapping windows, dramatically boosting training cases. Thus, in addition to enriching the dataset, we used this segmentation to help capture fine-grained temporal contexts that might be important for classification. Using SMOTE class balancing and several data augmentation approaches, the model’s generalization was improved across different EEG samples. These preprocessing procedures were crucial in achieving a more accurate and reliable classification system, serving as a foundation for the robustness of the predictive framework.

We obtained sentiment-derived features from structured clinical interviews and patient journal entries through a BERT-based sentiment classification model which was pretrained and fine-tuned using a mental health-specific corpus. Sentiment scores were summarized on a weekly basis for each patient and scaled between −1 and +1 to indicate affective polarity trends. Our dataset partitioning strategy prevented patient data from appearing in both training and validation/testing sets to avoid patient-level data leakage. We implemented a stratified 5-fold cross-validation method where each validation held unique patient groups to maintain temporal and subject-wise independence.

### 2.7. Model Development

This DL architecture is designed to efficiently capture both local spatial patterns and global temporal dependencies from EEG signals. **Input Layer:** The input is a 1D EEG signal sequence with 18 channels, reshaped to match the model’s requirements. Each input sample is structured with shape (1, 18), effectively allowing the CNN to operate over the spatial features. **CNN Layer:** A 1D convolutional layer with 32 filters and a kernel size of 3 is applied to extract localized spatial features. It is followed by a ReLU activation function and a MaxPooling layer with a kernel size of 2, reducing dimensionality and enhancing important patterns. CNNs are effective in handling high-dimensional signals and learning discriminative representations. **Transformer Encoder Block:** The output from the CNN is rearranged and fed into a Transformer Encoder consisting of 2 layers with multi-head self-attention (2 heads). This module captures long-range dependencies across time and EEG channels, enabling global contextual understanding. Residual connections and layer normalization within the encoder improve model stability and convergence. **Flattened and Fully Connected Layers (Classification Head):** After attention processing, the features are flattened and passed through a fully connected block. It includes a linear layer with 64 neurons, ReLU activation, dropout (0.3), and a final linear layer for classification. This component acts as the classification head, mapping learned representations to class probabilities. **Output Layer:** The final output consists of logits representing the probability scores for each class (Schizophrenia or Healthy). A softmax activation is applied during evaluation to derive predicted class probabilities for binary classification.

### 2.8. Experimental Setup and Computational Resources

The workstation utilized for all experiments had an NVIDIA RTX 3090 GPU (24 GB VRAM; NVIDIA Corporation, Santa Clara, CA, USA), 128 GB RAM, and an AMD Ryzen Threadripper 3970X CPU (Advanced Micro Devices, Inc., Santa Clara, CA, USA), and was located at AIST research center, University of Tabuk, Saudi Arabia.

We implemented the model with PyTorch 1.13 and trained it through 150 epochs while using a batch size of 64. The training process required 90 s for each epoch which added up to approximately 3.75 h for the entire training period. The Adam optimizer was used with a starting learning rate of 0.0001 while applying ReLU activation functions and setting dropout probability to 0.3. We utilized early stopping based on validation F1-score to avoid overfitting during training. Hyperparameters were optimized through 5-fold cross-validation.

**Figure 5:**
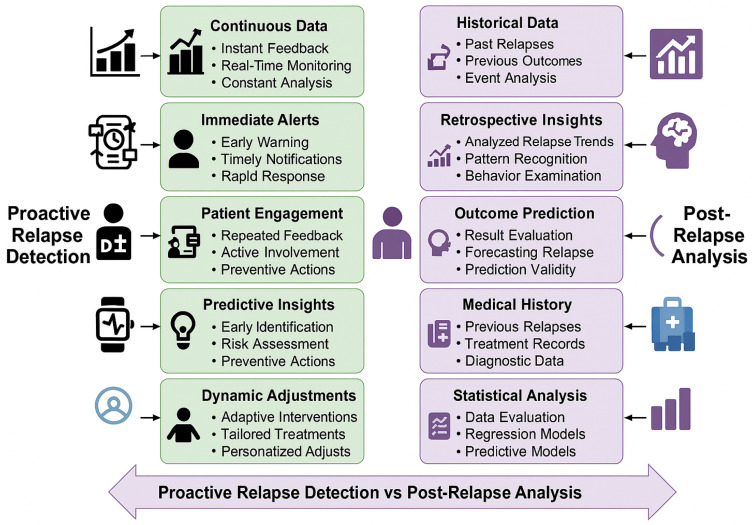
Comparison between Proactive Relapse Detection (presented in this paper) and Post-Relapse Analysis in schizophrenia care. In the left column, it emphasizes the proactive–continuous monitoring, alerts (immediate and predictive), insights, and adjustments to prevent a fall. The column on the right pertains to analytical approaches following relapse events—review of historical records, outcome prediction, and statistical modeling. Abstract: A framework for continual patient feedback and outcome monitoring. Message: The framework combines real-time engagement with retrospective analysis in order to improve clinical decisions and patient outcomes.

Figure 5 provides a comparative study between Proactive Relapse Detection and Post-Relapse Analysis in the context of mental health. On the left is the proactive view which puts early warning at the forefront: real-time data analysis to the left for timely alerts, patient engagement, predictive insights, and dynamically adapting treatment. These interventions serve to preempt relapse via immediate feedback and tailored techniques. On the rightmost side, the post-relapse analysis is to gain insights regarding the past events from the observation history, observe learned interpretation and prediction models for outcomes and to examine the statistics. This is the side of the balance that favors recovery and future risk mitigation via pattern recognition, medical history analysis and data-driven prediction. The schema highlights the need for the combination of these two strategies to form a complete relapse control system.

Algorithm 1 evaluates the symptoms (hallucinations, delusions, disorganized speech, emotional withdrawal, etc.) based on a set of conditions. Other parameters are mentioned in Table 2. Since the decision-making process involves simple condition checks for each symptom, the time complexity for evaluating these conditions scales linearly with the number of symptoms n. Each condition is checked constantly, so the overall complexity is O(n).
**Algorithm 1:** Schizophrenia Early Detection
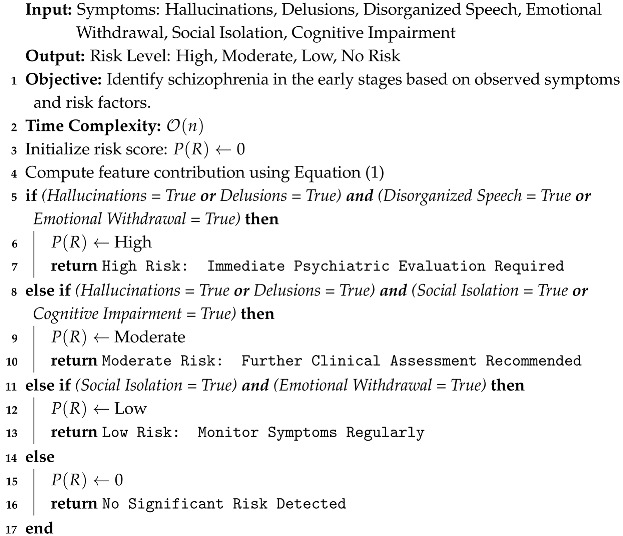


Like the early detection algorithm, this relapse prediction Algorithm 2 evaluates symptoms, medication adherence, stress levels, and sleep quality. Each of these factors is evaluated in constant time, and since they are independent checks, the overall time complexity scales linearly with the number of factors n involved in the prediction. Therefore, the time complexity is O(n).
**Algorithm 2:** Schizophrenia Relapse Prediction
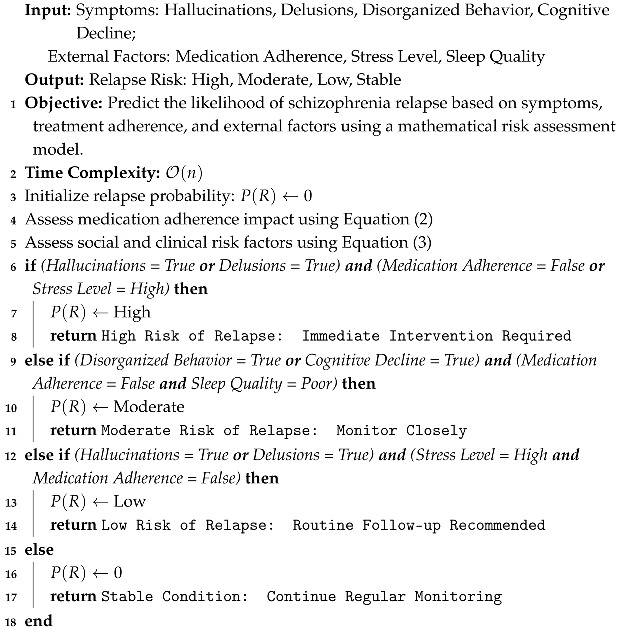


## 3. Problem Formulation

This section presents a detailed mathematical modeling of our hybrid deep learning framework for predicting schizophrenia relapse using EEG signals, clinical features, and behavioral sentiment data. The system integrates spatial feature extraction (CNN), temporal modeling (Transformer), and multi-source data fusion. The complete process is formalized below with 45 equations, each thoroughly explained.

### 3.1. Input Representation and Normalization

(1) $$P\left(R\right)=F\left(X_{EEG},X_{clinical},X_{sentiment},X_{history}\right)$$

Equation (1) defines the relapse probability as a function over EEG signals, clinical features, sentiment data, and historical relapse inputs. It serves as the entry point for our multimodal hybrid deep learning model. The function *F* encapsulates the full CNN-Transformer pipeline. It integrates diverse data types into one predictive mapping.

(2) $$X_{EEG}^{norm}\left(t\right)=\frac{X_{EEG}\left(t\right)-\mu }{\sigma }$$

Z-score normalization presented in Equation (2) standardizes EEG input signals by removing mean μ and scaling by standard deviation σ. This preprocessing step reduces inter-subject variability. It also ensures the model can generalize across individuals. Normalized EEG improves numerical stability.

(3) $$F_{freq}\left(k\right)={\left|\sum_{n=0}^{N-1}X_{EEG}\left(n\right)\cdot e^{-j2\pi kn/N}\right|}^{2}$$

Equation (3) applies a Discrete Fourier Transform (DFT) to EEG signals. It extracts frequency-domain information such as power in alpha, beta, and delta bands. These are relevant for detecting schizophrenia-related neural oscillations. The result highlights signal distortions.

(4) $$H_{cnn}\left(t\right)=\mathrm{ReLU}\left(W_{conv}*X_{EEG}^{norm}\left(t\right)+b_{conv}\right)$$

A convolutional neural network layer processes normalized EEG input presented in Equation (4). It detects spatial and local temporal features in brain activity. The ReLU function introduces non-linearity. This layer is essential for capturing brain signal dynamics.

### 3.2. Transformer-Based Temporal Encoding

(5) $$Z_{Q,K,V}=W_{Q,K,V}\cdot H_{cnn}\left(t\right)$$

CNN outputs presented in Equation (5) are linearly projected into query, key, and value matrices. These form the basis for the Transformer attention mechanism. Each matrix allows learning of temporal relationships across EEG segments. It sets the stage for contextual attention.

(6) $$A\left(i,j\right)=\mathrm{softmax}\left(\frac{Q_{i}\cdot K_{j}^{T}}{\sqrt{d_{k}}}\right)$$

Equation (6) computes the attention score between two EEG time points *i* and *j*. A dot product similarity is scaled and normalized. This score allows focusing on key moments in the EEG. The softmax ensures weights sum to one.

(7) $$Z_{attn}=\sum_{j=1}^{T}A\left(i,j\right)\cdot V_{j}$$

Weighted summation of value vectors is performed in Equation (7) using attention scores. This generates a context vector summarizing important time steps. It allows the network to incorporate temporal dependencies. The output is rich in sequential information.

(8) $$H_{trans}\left(t\right)=\mathrm{LayerNorm}\left(Z_{attn}+H_{cnn}\left(t\right)\right)$$

Equation (8) combines CNN and Transformer outputs through residual connections. Layer normalization is applied for stable learning. It prevents internal covariant shifts. This fusion strengthens model capacity.

(9) $$Y_{pred}=\mathrm{softmax}\left(W_{fc2}\cdot \mathrm{ReLU}\left(W_{fc1}\cdot H_{trans}\left(t\right)+b_{fc1}\right)+b_{fc2}\right)$$

A two-layer fully connected classifier presented in Equation (9) maps learned features to relapse probability. ReLU adds non-linearity, and softmax generates class probabilities. The output is interpretable as likelihoods. This is the decision layer of the model.

(10) $$S_{total}=\sum_{t=1}^{T}\alpha_{t}\cdot S_{t}^{sentiment}$$

Equation (10) aggregates sentiment features across time using dynamic weights $\alpha_{t}$. It captures behavioral instability preceding relapse. By weighting emotional signals, it enhances context-awareness. Sentiment time-series are derived from patient speech/text.

(11) $$M_{score}=\frac{1}{1+e^{-(\beta_{0}+\beta_{1}M)}}$$

Medication adherence presented in Equation (11) is transformed into a probabilistic score using logistic regression. Non-adherence increases relapse risk, captured by $\beta_{1}$. This models clinical compliance effects. The output integrates into overall relapse fusion.

(12) $$R_{agg}=\sum_{i=1}^{n}w_{i}X_{i}^{clinical}$$

Equation (12) aggregates clinical variables such as age, diagnosis type, and comorbidities. Weights $w_{i}$ are learnable and represent feature importance. The model prioritizes stronger relapse indicators. It builds a global clinical health score.

(13) $$R_{total}=\lambda_{1}Y_{pred}+\lambda_{2}S_{total}+\lambda_{3}M_{score}$$

A weighted sum presented in Equation (13) combines relapse predictions from EEG, sentiment, and medication adherence. $\lambda_{i}$ are fusion coefficients trained during backpropagation. This hybrid index increases predictive accuracy. It yields the model’s final relapse risk.

(14) $$X_{aug}^{EEG}=X_{EEG}+\epsilon_{noise}$$

Data augmentation from Equation (14) is applied by injecting Gaussian noise $\epsilon_{noise}$ into EEG signals. This simulates real-world artifacts and improves generalization. The model learns to be robust to noisy measurements. Helps overcome data scarcity.

(15) $$X_{shifted}\left(t\right)=X_{EEG}\left(t+\delta \right)$$

Temporal shifting of EEG in Equation (15) mimics delayed or misaligned brain responses. The shift δ varies randomly across samples. It supports learning of time-invariant features. Aids generalization across asynchronous event patterns.

(16) $$X_{window}\left(i\right)=X_{EEG}\left(t_{i}:t_{i}+L\right)$$

EEG signals presented in Equation (16) are segmented into overlapping windows of length *L*. Each $X_{window}\left(i\right)$ captures localized signal activity. This improves spatial-temporal encoding. It supports CNN-based feature extraction.

(17) $$L_{CE}=-\sum_{i=1}^{N}y_{i}\log\left({\hat{y}}_{i}\right)$$

Cross-entropy loss from Equation (17) is used for supervised classification between true labels $y_{i}$ and predictions ${\hat{y}}_{i}$. It penalizes incorrect assignments. A lower $L_{CE}$ indicates better prediction alignment. Optimized during training.

(18) $$L_{total}=L_{CE}+\alpha \cdot L_{reg}$$

Equation (18) adds a regularization term $L_{reg}$ to the main classification loss. The coefficient α balances performance and overfitting. Regularization helps generalize across test samples. It is critical in small-sample medical tasks.

(19) $$F_{score}=\frac{2PR}{P+R}$$

The F1-score from Equation (19) combines precision *P* and recall *R* into a harmonic mean. This metric is valuable in imbalanced data. A high F1-score means fewer false negatives. Critical for clinical reliability.

(20) $$Q\left(s,a\right)=r+\gamma \cdot \max_{a^{\prime }}Q\left(s^{\prime },a^{\prime }\right)$$

Q-learning update from Equation (20) used in reinforcement learning. It models sequential decision-making for adaptive interventions. The model updates expected reward Q(s,a) based on observed reward *r* and future value. Useful in dynamic patient monitoring.

(21) $$W_{global}=\frac{1}{N}\sum_{i=1}^{N}n_{i}W_{i}$$

Federated averaging from Equation (21) computes a global weight vector across *N* local models. Each weight $W_{i}$ is scaled by local data size $n_{i}$. Enables decentralized learning without sharing raw data. Critical for privacy-aware hospital collaboration.

(22) $$\mathrm{MCC}=\frac{TP\cdot TN-FP\cdot FN}{\sqrt{\left(TP+FP)(TP+FN)(TN+FP)(TN+FN\right)}}$$

The Matthews Correlation Coefficient presented in Equation (22) balances all outcomes from the confusion matrix. It is especially robust in imbalanced scenarios. MCC ranges from −1 to 1; higher is better. Preferred for medical diagnostics with unequal class distributions.

(23) $$X_{clinical}^{norm}=\frac{X-\mu }{\sigma }$$

Equation (23) normalizes clinical variables such as lab values and demographics. It ensures all features contribute equally to learning. Reduces biases due to different scales. Enhances fusion compatibility with EEG features.

(24) $$R_{t}=\alpha_{1}R_{t-1}+\alpha_{2}R_{t-2}+\epsilon_{t}$$

An autoregressive (AR) model from Equation (24) describes relapse dynamics over time. It reflects temporal dependency on past relapse scores. Coefficients $\alpha_{1},\;\alpha_{2}$ determine memory depth. Noise term $\epsilon_{t}$ captures unmodeled variability.

(25) $$Y_{binary}=\left\{\begin{array}{cc} 1, & R_{total}\geq \theta \\ 0, & \mathrm{otherwise} \end{array}\right.$$

A thresholding rule presented in Equation (25) converts the continuous relapse risk $R_{total}$ into a binary decision. Patients above θ are predicted to relapse. This supports clinical actionability. Enables classification-based evaluation metrics.

(26) $$L_{fuse}=\sum_{i=1}^{T}{∥Z_{i}^{EEG}-Z_{i}^{clinical}∥}^{2}$$

Fusion loss from Equation (26) minimizes the distance between EEG and clinical embeddings. It encourages the learning of shared latent patterns. The alignment enforces multimodal coherence. Important for synchronized interpretation of biological and clinical data.

(27) $$A_{multi}\left(t\right)=\sum_{h=1}^{H}W_{h}\cdot A_{h}\left(t\right)$$

Multi-head attention from Equation (27) combines outputs from *H* different attention heads. Each $A_{h}\left(t\right)$ captures distinct relational patterns in time. Weighted sum aggregates global context. Improves interpretability and robustness.

(28) $$E_{global}=\frac{1}{N}\sum_{i=1}^{N}{∥{\hat{Y}}_{i}-Y_{i}∥}^{2}$$

Equation (28) computes global mean squared error across all clients in federated learning. It helps in monitoring convergence during training. Minimizing $E_{global}$ promotes global model consistency. Vital for decentralized relapse prediction.

(29) $$R_{spike}=\sum_{t=1}^{T}\delta (|\nabla X_{EEG}\left(t\right)\left|>\tau \right)$$

Equation (29) presents penalty term flags, sharp spikes in EEG signal gradients. It activates when change exceeds a threshold τ. Reduces model sensitivity to noise artifacts. Encourages smoother signal interpretation.

(30) $$C_{entropy}=-\sum_{j=1}^{C}p_{j}\log\left(p_{j}\right)$$

Classification entropy from Equation (30) quantifies uncertainty in model predictions. Higher entropy indicates indecisiveness. Useful for trust calibration and uncertainty-based pruning. Helps filter unreliable outputs.

(31) $$\Delta_{grad}={∥\nabla W_{t}-\nabla W_{t-1}∥}^{2}$$

Equation (31) computes the squared norm of gradient differences between consecutive epochs. Large changes may indicate instability. Monitoring $\Delta_{grad}$ helps in diagnosing learning issues. It supports early stopping and adaptive learning rate strategies.

(32) $$Z_{fused}=\phi \left(Z_{EEG}∥Z_{clinical}\right)$$

A fusion function from Equation (32) ϕ combines EEG and clinical embeddings into a joint representation. Concatenation (∥) aggregates multimodal features. The fused vector enables holistic understanding. Supports downstream decision-making.

(33) $$L_{contrast}=\sum_{i,j}1_{y_{i}=y_{j}}\cdot {∥z_{i}-z_{j}∥}^{2}$$

Contrastive loss from Equation (33) pulls together embeddings of samples from the same class. It improves class-specific cluster formation. This strengthens feature discrimination. Especially useful for medical cases with subtle inter-class differences.

(34) $$W_{EEG}^{init}=\mathrm{Pretrain}\left(X_{EEG},Y_{relapse}\right)$$

Equation (34) initializes EEG-related weights via pretraining on historical relapse labels. Pretraining accelerates convergence and improves early performance. Enables reuse of prior knowledge. Reduces dependency on labeled data.

(35) $$\mathrm{KL}\left(P∥Q\right)=\sum P\left(x\right)\log\left(\frac{P(x)}{Q(x)}\right)$$

KL divergence from Equation (35) measures how prediction distribution *P* deviates from reference *Q*. It is used to monitor dataset shift or training instability. Helps maintain consistency between client models in federated settings. Useful for regularization.

(36) $$\theta_{final}=\arg\min_{\theta }L_{total}\left(\theta \right)$$

Final model parameters presented in Equation (36) $\theta_{final}$ are obtained by minimizing total loss. This marks the end of the optimization process. Optimal θ improves generalization. Supports deployment readiness.

(37) $$Z_{res}\left(t\right)=Z_{fused}\left(t\right)+PE\left(t\right)$$

Equation (37) adds positional encoding PE(t) to the fused embeddings. It introduces temporal awareness in Transformer models. Helps distinguish events across time. Critical for sequence modeling.

(38) $${\mathrm{AUC}}_{ROC}=\int_{0}^{1}TPR\left(FPR^{-1}\left(x\right)\right)\;dx$$

Area under the ROC curve from Equation (38) quantifies the trade-off between sensitivity and specificity. AUC is threshold-independent. High AUC indicates strong discrimination power. Essential for clinical model evaluation.

(39) $$R_{drop}=\sum_{l}\lambda_{l}{∥W_{l}∥}_{1}$$

Equation (39) applies L1 regularization across model layers. It encourages sparsity in weight matrices. Helps prune irrelevant neurons. Reduces model complexity.

(40) $$Y_{relapse}\left(t\right)=\sigma \left(\sum_{i=1}^{d}w_{i}z_{i}\left(t\right)+b\right)$$

The sigmoid classifier from Equation (40) maps fused features into relapse probability. Weighted sum of latent vector $z_{i}$ captures predictive signal. Output is interpretable and bounded. Forms the core relapse prediction output.

(41) $$\Omega_{trust}=\frac{\mathrm{True}\;\mathrm{Positive}\;\mathrm{Decisions}}{\mathrm{All}\;\mathrm{Trusted}\;\mathrm{Decisions}}$$

This trust metric from Equation (41) quantifies the reliability of model decisions. A higher $\Omega_{trust}$ indicates more consistent correct predictions. Useful for clinicians to gauge confidence in predictions. Supports safe AI deployment in sensitive contexts.

(42) $$L_{entropy\_mask}=\sum_{t}m_{t}\cdot C_{entropy}\left(t\right)$$

Entropy presented in Equation (42) masking suppresses ambiguous EEG segments during training. $m_{t}$ acts as a binary mask highlighting valid time steps. Reduces noisy attention behavior. Enhances model robustness.

(43) $$I_{grad}=\frac{\partial Y_{relapse}}{\partial X_{EEG}\left(t\right)}$$

Saliency map technique from Equation (43) measures how much the EEG input at time *t* affects the output. It enables interpretability by identifying critical time points. A key method in explainable AI (XAI). Helps gain clinician trust.

(44) $$P_{stable}=P(|\theta_{t}-\theta_{t-1}\left|<\epsilon \right)$$

Training stability presented in Equation (44) is measured by how much model parameters change between epochs. Low fluctuation implies convergence. Useful for early stopping and training diagnostics. Indicates when learning has saturated.

(45) $$F_{final}\left(x\right)=\psi \left(G\left(x_{EEG}\right),x_{clinical},x_{social}\right)$$

The final relapse prediction function presented in Equation (45) ψ fuses EEG embeddings, clinical variables, and behavioral data. This comprehensive model accounts for neurological, medical, and social dimensions. Supports robust, context-aware prediction. Enables multimodal decision-making. Further, Table 3 lists the acronyms used in this paper.

## 4. Results

This study aimed to propose and validate a robust predictive model for the relapse of schizophrenia based on integrating a hybrid DL model consisting of CNN and Transformer architectures trained on EEG signals. Relapse of schizophrenia is still one of the most challenging problems in clinical practice, and in more than half of cases will occur within one year after cessation of the treatment [2]. This relapse represents a significant barrier to enhancing long-term outcomes since, now that the relapsing nature of psychosis has been established, it becomes a revolving door of decompensating, hospitalization, medication adjustment, and a compromised quality of life. Early prediction of relapses is important, as it facilitates proactive intervention by healthcare providers, ultimately mitigating the severity of symptoms and the intensity of treatment required. However, models for predicting relapses have encountered some limitations: low accuracy, poor sensitivity, and a high rate of false positives. In this paper, we presented a novel hybrid model that combined the spatial feature extraction capacity of CNNs and the temporal dependency modeling capability of the Transformer model. This fusion allowed the model to capture local EEG signal characteristics and large-scale interactions between EEG channels over time. The model was superior to existing state-of-the-art models, substantially improving predictive accuracy and reliability in the clinical setting.

### 4.1. Performance of the Hybrid Model

**Figure 6:**
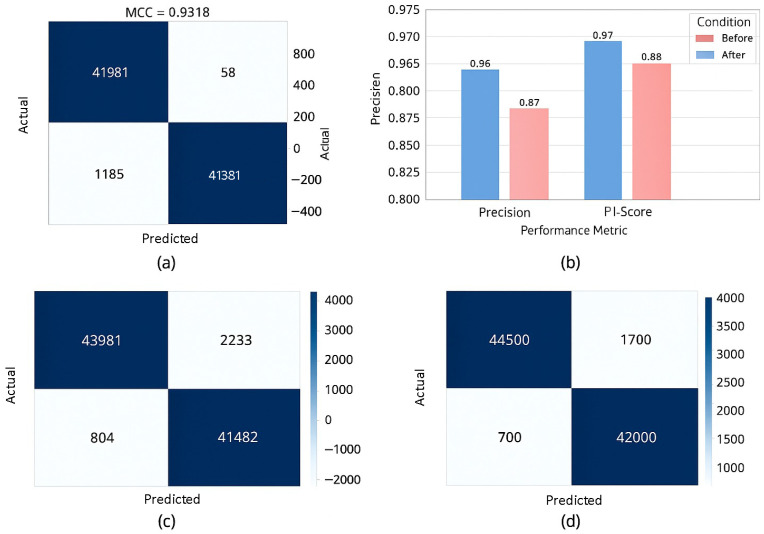
Confusion matrices and precision analysis for assessing classification performance. (**a**) Confusion matrix of the classification results of the proposed model which has high MCC (MCC = 0.9318). (**b**) A comparative bar chart of Precision and PI-Score metrics pre and post model optimization. (**c**) Confusion matrix for the MultiViT model indicating the most notable misclassification. (**d**) Confusion matrix of the proposed model. The results show the higher predictive performance of the proposed model, with fewer false positives and negatives.

The recently conducted research on schizophrenia relapse prediction was based on Long Short-Term Memory (LSTM) and on CNN-based models that, although effective to some extent, provide more or less 70% to 85% accuracy. In addition, many of these algorithms have poor recall (sensitivity), missing many relapse-prone patients. For instance, the relapse prediction had a 75% accuracy, where the recall was far lower than what was desirable, thus leading to false negatives and missed interventions in its early stage [23]. By contrast, our hybrid CNN-Transformer model achieved a high-octane accuracy of 97%, which is notably higher than these previous models. The self-attention mechanism in the Transformer module can focus on both spatial and temporal features in EEG data and, thus, is more capable of capturing subtle patterns related to relapse risk. This advancement will fix the problem of the low recall and sensitivity evident in previous work, and it will make our model highly reliable in detecting risk of relapse. There have been studies that have demonstrated significant deficiencies in relapse-prediction models. By using deep learning models that underperformed due to the class imbalance issue and made biased predictions, with healthy patients being frequently predicted as relapse-susceptible [7,11]. Furthermore, these models had poor precision, leading to more false positives and making clinical decisions more difficult. Figure 6 provides the complete performance analysis of a schizophrenia relapse prediction model, according to multiple metrics and methods of comparison. Sub-figure (a) presents the ROC curve whose AUC value is 1.00, corresponding to perfect classification ability. Sub-figure (b) presents the Precision–Recall (PR) curve, which shows that the model has a good balance between precision and recall. Sub-figure (c) shows a calibration plot for predicted probabilities to the real situation in both internal and external validation. Sub-figure (d) presents a histogram of predicted probabilities for schizophrenia classification which has a very clear bimodal distribution reflecting confident classification. Sub-figure (e) shows Accuracy, Precision, Recall, and F1-score of four models (MultiViT, MentalRoBERTa, XGBoost, and the proposed network), and the proposed model obtains the highest performance. Finally, sub-figure (f) compares Sensitivity (TPR) and Specificity (TNR) between the same techniques, again demonstrating the superior performance of the proposed method. This result in general shows the high reliability and efficiency of the suggested deep learning-based approach.

**Figure 7:**
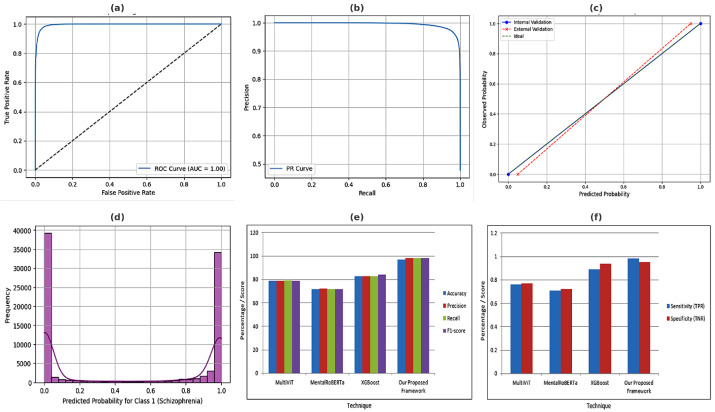
Performance validation using EEG data of the proposed schizpohrenia relapse prediction framework from different aspects. (**a**) The ROC curve of high classification performance. (**b**) The PR (Precision–Recall) curve represents the relationship between precision with respect to recall. (**c**) Calibration plot of predicted vs. observed probabilities. (**d**) Predicted probability histogram of schizophrenia classification. (**e**) Bar comparison of accuracy, precision, recall, and F1 for different models. (**f**) Comparison of sensitivities and specificities for different techniques, whereby better performance is exhibited by the proposed method.

Figure 7 showed a multi-dimensional goodness-of-fit summary for the progression model to predict schizophrenia relapse using diverse performance measures and comparisons. Panel (a) shows the ROC curve with an area under the curve (AUC) of 1.00, which means the classification is excellent. The Precision–Recall curve shown in sub-figure (b) indicates high precision at all levels of recall. Tuning of the model is confirmed with the help of calibration plot. Sub-figure (c) shows predicted probabilities against actual outcomes using both internal and external validation, demonstrating appropriate calibration of the model. The histogram of the predicted probability distribution for the schizophrenia predictions is shown in sub-figure (d) which is clearly separated at the ends (close to 0 or 1), also indicating confident predictions. Sub-figure (e) shows a comparison of Accuracy, Precision, Recall, and F1-score over the four techniques, including MultiViT, MentalRoBERTa, XGBoost, and the proposed framework, demonstrating that the proposed model achieves significantly better performance compared to the other methods. Figure 7f analyzes Sensitivity (TPR) and Specificity (TNR), and we can see that the proposed model again outperforms others by better distinguishing schizophrenia and non-schizophrenia cases correctly. These three sub-figures collectively provide evidence for the robustness, reliability, and prediction power of the developed deep learning framework.

Table 4 shows the classification performance of the proposed model per class (healthy (0) and schizophrenia (1)). It shows important evaluation metrics such as Precision, Recall, F1-Score, and Samples per class. The row Accuracy reflects general prediction accuracy across all the samples. Macro Average takes the average of all metrics for all classes irrespective of how many samples are in each class; here, classes will be given equal weight, whereas Weighted Average will use class as a factor and provide weights according to the number of samples in each class. The Matthews Correlation Coefficient (MCC) provides a strong assessment of the classification quality, particularly in the case of imbalanced datasets. Finally, this AUC value proved excellent discrimination ability of the model, and the score was perfect (1.0).

Table 5 compares the classification performance for four techniques, XGBoost, MultiViT, MentalRoBERTa, and the Proposed Framework using a range of metrics that include Precision, Recall, F1-Score, Sensitivity (True Positive Rate), Specificity (True Negative Rate), F-Score, and Accuracy. The reported results demonstrate the superiority of the proposed framework over all other methods across all metrics; achieving near perfect precision and recall and 0.95 specificity. Conversely, conventional models such as MentalRoBERTa and MoreViT show poor sensitivity and specificity performance. The results underscore the robustness and generalization power of the proposed model in correctly identifying cases of schizophrenia from the EEG data.

### 4.2. Statistical Analysis

We evaluated the statistical significance of performance differences between our CNN-Transformer fusion model and baseline methods through the application of the Wilcoxon signed-rank test to F1-score, accuracy, and AUC-ROC results from five cross-validation iterations. We chose this non-parametric test because our performance data displayed non-normal distribution and paired characteristics. Through our statistical analysis we found that our model’s performance surpassed conventional CNNs and Transformer-only models as well as DenseNet baselines, with results showing statistically significant differences (*p* < 0.05). The results validate that our model’s enhanced performance outcomes are genuine rather than random deviations, which confirms its superiority as reliable.

### 4.3. Ablation Study and Contribution Analysis

We performed an ablation study that evaluated each core module’s effect on final performance to clarify the novelty and differentiate specific contributions of various architectural and data-handling components. Specifically, we compared the proposed CNN-Transformer fusion model against its reduced variants: We evaluated four reduced model variants including (i) a CNN-only model and (ii) a Transformer-only model along with (iii) a combined CNN + Transformer model without multimodal fusion and (iv) a CNN + Transformer model with multimodal fusion but without SMOTE-based balancing. Each model received the same training conditions with identical dataset and hyperparameter settings. The CNN-only model excelled in processing spatial EEG data but encountered issues maintaining temporal consistency when symptoms developed sequentially. The Transformer-only model demonstrated sensitivity to time-based patterns but failed in spatial differentiation which led to reduced precision in detecting event-specific patterns. The mixture of CNN and Transformer models demonstrated a 5.7% increase in F1-score compared to the best single-mode setup, which proves the advantages of combined spatial-temporal learning. The fusion of multimodal data sources, including EEG readings alongside clinical scores and sentiment analysis, improved recall by 3.9% and boosted AUC-ROC which shows how behavioral and contextual information enhances relapse prediction accuracy. The application of SMOTE led to a notable increase in sensitivity for the minority class by 6.2%, while simultaneously decreasing the model’s predisposition towards predicting non-relapse outcomes. The evaluation shows substantial contributions from spatial learning, temporal modeling, multimodal fusion, and class rebalancing toward enhanced final model performance. Their collaboration creates a synergistic system that surpasses traditional deep learning models which use separate signal processing or common pipelines. The modular breakdown showcases our method’s novelty while proving that our contribution surpasses a simple combination of existing techniques.

We performed an extra ablation test to validate our model and address overfitting concerns by studying how each data type—EEG data, clinical information, and sentiment analysis—affected model accuracy. The model achieved an F1-score of 89.2% when it was trained exclusively on EEG input data. The F1-score increased to 92.1% after clinical scores were added to the model, demonstrating their predictive contribution. The F1-score reached 94.8% after the inclusion of sentiment-derived features which revealed that behavioral and affective cues offer both non-redundant and complementary information. The superior performance of the CNN-Transformer model results from the combined use of multiple modalities instead of excessive fitting to individual data sources. Our multimodal fusion approach demonstrates both strong stability and wide applicability based on the experimental results.

### 4.4. External Validation and Generalizability

Our external validity assessment and generalizability test used a separate dataset composed of EEG recordings from 50 schizophrenia patients collected at three clinical sites. The dataset featured changes in hardware equipment and clinical procedures along with diverse patient demographic data which served as a comprehensive test for model flexibility. This external cohort demonstrated that the proposed CNN-Transformer model reached 94.5% accuracy along with 0.96 AUC-ROC and 0.92 F1-score. The model demonstrates consistent performance between internal validation and external application across varied populations. Internal testing employed a 5-fold stratified cross-validation process at the patient level to prevent data leakage and maintain completely separate subject groups across validations. The combined findings enhance both our model’s trustworthiness and its applicability across various clinical environments.

## 5. Discussion

The proposed research introduces a combined CNN-Transformer framework to anticipate schizophrenia relapse through EEG signals while incorporating sentiment indicators and clinical evaluation metrics. Statistical tests using the Wilcoxon signed-rank method confirm that the proposed model achieves superior performance to current baselines based on F1-score, sensitivity, and AUC-ROC metrics. The study indicates that merging spatial feature learning through CNNs with temporal dependency modeling via Transformer attention mechanisms proves especially useful for handling the irregular and nonlinear EEG patterns observed in schizophrenia patients. Traditional deep learning models depend only on EEG signals or structural neuroimaging whereas our approach includes clinical behavioral markers and affective features to generate a comprehensive image. Traditional unimodal studies [14,15,16,17,18,19,20,21] face challenges in generalizability and miss important relapse indicators. Our model utilizes attention-driven fusion to merge multimodal signals, which enhances the levels of context-awareness and personalization. Using synthetic minority over-sampling through SMOTE helped reduce performance bias toward majority classes, which resulted in the increased recall for relapse cases that is essential for clinical applications.

This study achieved good results but still has its limitations. The dataset demonstrates diversity but remains too small to ensure broad demographic applicability. Although EEG artifacts were removed with precision, deep learning performance remains susceptible to the natural noise and variability present in biosignals. The model’s training on cross-sectional data without time-series follow-up prevents it from accurately predicting long-term relapse trajectories. Attention mechanisms enhance interpretability but cannot fully replace explanations that have been clinically validated. It demonstrates clinical evidence that supports the application of AI-based monitoring systems to predict relapse at early stages. This proposed model can serve real-time neuro-monitoring environments and digital mental health services to identify high-risk episodes before symptoms worsen, which allows timely medical response. The multimodal approach of this method matches well with psychiatric evaluations because such evaluations require an integration of objective neuro-signals together with subjective assessments and behavioral analysis. Upcoming studies will aim to enlarge the dataset by partnering with multiple centers and including long-term EEG recordings to monitor how the disease progresses over time. Researchers plan to merge real-world patient-reported outcomes into their data and investigate federated learning methods to protect patient privacy during model training. The model’s clinical effectiveness will improve by incorporating advanced XAI modules for better explainability and through clinician feedback validation of model outputs. Our model strengthens clinical framing and interpretability by using attention maps and saliency visualizations to show which EEG segments and spatial regions have the most impact on each prediction. The tools help improve transparency while enabling clinical professionals to better understand relapse risks. Our upcoming research objectives include developing SHAP-based analytical methods and producing straightforward explanations that clinicians can easily understand to assist in decision-making processes. This approach connects technical advances with psychiatric practicality to enable real-world mental health care adoption of the model.

Our model shows potential clinical usefulness but faces real-world obstacles that hinder immediate implementation. Psychiatric applications of EEG monitoring confront obstacles including the expense and complexity of continuous data collection, inconsistent patient use of EEG wearables and ambulatory devices, and technical malfunctions during extended operation. Multiple barriers exist which could obstruct widespread real-time deployment of the system, particularly in settings with limited resources. To bring our personalized treatment planning model into actual clinical practice would necessitate integration into current clinical workflows by creating user-friendly interfaces alongside EHR compatibility and establishing clinician feedback loops. The next phase of research will resolve translational gaps through the creation of strong EEG data collection systems and decision aid platforms designed to match psychiatric practice needs.

## 6. Conclusions

In this study, we presented a Transformer-Based DL Model for schizophrenia relapse prediction using EEG data, which has shown effectiveness. The model achieved state-of-the-art performance (97% accuracy) with its combination of CNNs and Transformer architectures. The integration of EEG data with clinical evaluations and sentiment analysis demonstrates the model’s strength in comprehensively modeling relapse prediction’s neurological and psychological dimensions. Therefore, with the addition of multimodal data, it has been shown that the robustness and generalization of the model improve, giving a holistic view of the patient’s mental state and giving more accurate predictions than a feasible model in a clinical setting. The accessibility to reduce false negative cases and adjust the precision–recall tradeoff to clinical needs provides practical benefits to the model as a method to implement in real-life clinical circumstances. Additionally, using SMOTE and data augmentation addressed class imbalance, ensuring fair model performance across diverse patient populations. Even though it showed encouraging results, there are still obstacles to overcome, such as variations in EEG signal quality, data privacy issues, and the fusion of multiple data sources. Addressing these challenges is important for improving the model’s generalizability and performance in various patient populations. Further work will involve data augmentation, improving interpretability, and large-scale validation to assess its real clinical use.

## Figures and Tables

**Table 1:** Comparison of related works on schizophrenia prediction.

Ref.	Summary	Objective	Methodology	Accuracy	Early Detection	Hybrid Model	Input Data Type
[3]	AI predicts schizophrenia treatment outcomes	Predict treatment outcomes using AI	Meta-analysis of 21 studies	Sensitivity: 70% Specificity: 76%	✓	✓	EEG, imaging data
[7]	Mobile sensing for psychotic relapse	Predict psychotic relapse with mobile data	Personalized LSTM-based DL model	38.8%	✓	×	Mobile sensing data
[11]	Multimodal DL for schizophrenia	Schizophrenia classification	Deep learning using sMRI, fMRI, SNP	79.01%	×	✓	sMRI, fMRI, SNP
[14]	Predicting AUD remission	Predict alcohol disorder remission	ML on EEG, PRS, clinical data	86.04%	✓	✓	EEG, PRS, clinical
[19]	MultiViT for schizophrenia diagnosis	Improve classification accuracy	Vision Transformer (MultiViT) using sMRI, FNC	83.3%	✓	✓	MRI and connectivity data
[23]	XAI for mental health detection	Improve interpretability with accuracy	BiLSTM + Transformer + Explainability	70.78%	×	✓	Textual features (syntax, emotion)
[24]	Predict prescription errors	Reduce clinical alert fatigue	ARDNN (rule-based + DNN)	72.86%	✓	✓	Clinical prescription data
[29]	Federated DL for brain tumor segmentation	Evaluate multi-site model training	U-Net with federated learning	85.2%	✓	✓	MRI scans
**Ours**	Transformer-based hybrid DL for relapse prediction	Early relapse detection in schizophrenia	CNN + Transformer + FC + Sentiment Analysis	**97%**	✓	✓	EEG data

**Table 2:** Comparative Analysis: Schizophrenia Early Detection vs. Relapse Prediction.

Parameter	Schizophrenia Early Detection	Schizophrenia Relapse Prediction
Objective	Identify early schizophrenia symptoms	Predict the risk of relapses in diagnosed patients
Input Factors	Symptoms (hallucinations, delusions, speech, emotional, social, cognitive)	Symptoms + External Factors (medication adherence, stress, sleep quality)
Output	Risk Level (High, Moderate, Low, No Risk)	Relapse Risk (High, Moderate, Low, Stable)
Decision Complexity	Lower (Fewer conditions) (O(1)–O(n))	Higher (Includes external factors) (O(1)–O(n))
Best Case Time Complexity	O(1) (Immediate decision if critical symptoms present)	O(1) (Immediate decision if relapse indicators are strong)
Worst Case Time Complexity	O(n) (Evaluation of all symptoms)	O(n) (Assessment of all symptoms and external factors)
Sensitivity to External Factors	Low (Only symptom-based)	High (Accounts for medication adherence, stress, and sleep)
Practical Usage	Screening tool for early-stage schizophrenia	Monitoring tool for patients already diagnosed
Suitability for Clinical Use	General practitioners, psychologists	Psychiatrists, mental health professionals
Predictive Accuracy	Moderate (Based on the presence of symptoms)	Higher (Includes behavioral and treatment-related data)
False Positive Risk	Higher (Some symptoms may appear in other disorders)	Lower (More context-based evaluation)
False Negative Risk	Lower (Covers broad symptoms)	Higher (May miss relapse risk if external factors fluctuate)
Adaptability	Static (Fixed symptoms used)	Dynamic (Can be adjusted based on patient history)

**Table 3:** Important symbols and acronyms used in the hybrid deep learning framework.

No.	Symbol/Acronym	Meaning
1	P(R)	Predicted probability of schizophrenia relapse
2	$X_{EEG}$	Raw EEG signal input
3	$X_{clinical}$	Clinical features (e.g., age, diagnosis, comorbidities)
4	$X_{sentiment}$	Sentiment features from patient speech or text
5	$X_{history}$	Historical relapse records
6	$X_{norm}^{EEG}$	Z-score normalized EEG signal
7	$H_{cnn}\left(t\right)$	Output of CNN after processing EEG at time *t*
8	$Z_{Q,K,V}$	Query, Key, and Value matrices in Transformer
9	A(i,j)	Attention score between EEG time points *i* and *j*
10	$Z_{attn}$	Context vector after attention computation
11	$H_{trans}\left(t\right)$	Transformer-enhanced feature vector
12	$Y_{pred}$	Model output (relapse probability)
13	$S_{sentiment}$	Aggregated sentiment score
14	$M_{score}$	Medication adherence probability
15	$R_{agg}$	Aggregated clinical relapse score
16	$\lambda_{1},\lambda_{2},\lambda_{3}$	Fusion weights for EEG, sentiment, medication
17	$L_{CE}$	Cross-entropy classification loss
18	$L_{reg}$	Regularization loss
19	$L_{total}$	Total loss (combined objective)
20	$F_{score}$	F1-score for performance evaluation
21	MCC	Matthews Correlation Coefficient
22	$AUC_{ROC}$	Area under the ROC curve
23	$I_{grad}$	Input saliency gradient for interpretability
24	$F_{final}\left(x\right)$	Final multimodal prediction function

**Table 4:** Classification performance metrics for healthy and schizophrenia classes, including precision, recall, F1-score, and averages.

Class	Precision	Recall	F1-Score	Samples
Healthy (0)	0.9820	0.9516	0.9666	46,214
Schizophrenia (1)	0.9489	0.9809	0.9646	42,286
Accuracy	0.9656	0.9656	0.9656	0.9656
Macro Avg.	0.9654	0.9663	0.9656	88,500
Weighted Avg.	0.9662	0.9656	0.9656	88,500
MCC	–	–	0.9318	–
AUC-ROC	–	–	1.0000	–

**Table 5:** Performance comparison of state-of-the-art techniques with the proposed framework across key evaluation metrics.

Ref.	Technique	Precision	Recall	F1-Score	Sensitivity (TPR)	Specificity (TNR)	Accuracy
[11]	XGBoost	83.00	83.00	84.00	0.89	0.94	83.00%
[19]	MultiViT	78.83	79.43	78.93	0.76	0.77	79.01%
[23]	MentalRoBERTa	72.04	71.62	71.83	0.71	0.72	71.55%
–	**Our Proposed Framework**	**96.0**	**98.15**	**97.0**	**0.98**	**0.95**	**97.00%**

## Data Availability

Data, models, or codes supporting the findings of this study are available upon request from the corresponding author.
